# Design, Experimental and Numerical Characterization of 3D-Printed Porous Absorbers

**DOI:** 10.3390/ma12203397

**Published:** 2019-10-17

**Authors:** Tobias P. Ring, Sabine C. Langer

**Affiliations:** TU Braunschweig, Institute for Acoustics, 38106 Braunschweig, Germany; s.langer@tu-braunschweig.de

**Keywords:** porous material, biot model, additive manufacturing, inverse parameter identification, finite-element method, material design

## Abstract

The application of porous materials is a common measure for noise mitigation and in room acoustics. The prediction of the acoustic behavior applies material models, among which most are based on the Biot-parameters. Thereby, it is expected that, if more Biot-parameters are used, a better prediction can be obtained. Nevertheless, an estimation of the Biot-parameters from the geometric design of the material is possible for simple structures only. For common porous materials, the microstructure is typically unknown and characterized by homogenized quantities. This contribution introduces a methodology that enables the design and optimization of porous materials based on the Biot-parameters and connects these to microscopic geometric quantities. Therefore, artificial porous materials were manufactured using 3D-printing technology with a prescribed geometric design and the influence of different design variables was investigated. The Biot-parameters were identified with an inverse procedure and it can be shown that different Biot-parameters can be influenced by adjusting the geometric design variables. Based on these findings, a one-parameter optimization procedure of the material is set up to maximize the absorption characteristics in the frequency range of interest.

## 1. Introduction

The application of porous media in sound fields is a widely applied measure for noise mitigation. Thereby, two main applications are common. The first application is the use of porous absorbers and resonators to introduce acoustic damping into sound fields. This is widely applied in room acoustics in order to adjust the reverberation time of the room. In this field, resonators are mainly applied in the low frequency regime and for specific, narrow frequency bands. On the other side, porous absorbers are applied in the mid and high frequency regime, as the absorption performance of these materials basically increases with the frequency. The use of both, resonators and porous absorbers, is widely investigated and well understood, from the vast field of publications on this topic see, e.g., [1,2,3,4]. Another application of porous media is the reduction of noise generation in flows, i.e., in the field of aeroacoustics. Whereas porous liners oftentimes are applied to gas turbine aircraft engines with the goal of absorption (e.g., [5,6,7]), a relatively new field is the application of porous liners for reducing the flow noise generation. For the acoustics of aircraft, one of the major airframe noise sources is the noise generation at the aircraft wing’s trailing edge. The work presented in [8,9,10,11] shows the effect and investigates the application of porous media to mitigate the noise generation. Within the Collaborative Research Center (CRC) 880 at TU Braunschweig, several investigations have been conducted to reduce the aeroacoustic noise at the trailing edge by manufacturing the trailing edge of porous material. Basic design recommendations for this application are given in [12] and a manufacturing process for trailing edges with spatially varying material parameters is presented in [13]. In [14,15], numerical investigations are carried out that investigate the influence of porous liners that are placed on the outer skin of an aircraft on the structure borne sound energy flow into the airframe under an aeroacoustic load. It can be shown that the application of porous material reduces the sound energy that is transmitted into the structure and, hence, the vibration of the airframe is reduced. An investigation regarding the long-term application of porous media is done in [16]. This work covers the effect of degradation of the porous material due to fouling and staining and identifies the change of the material parameters during the degradation process.

The investigations mentioned above are just a small and far from exhaustive overview of applications for porous materials in acoustics. To achieve a sufficient performance, absorbers have to be designed and produced according to the design requirements. Nevertheless, the design and manufacturing of porous materials is a challenging task. The absorption characteristics highly depend on the microstructure of the materials such as pore geometry and pore size, as these properties influence macroscopic properties such as flow resistivity and surface impedance. For noise mitigation and reverberation time adjustment in interior applications, fibrous porous media such as mineral wool or melamine foams are widely applied. These materials incorporate good absorption characteristics combined with low interaction with the surrounding environment. On the other side, their microstructure is basically unknown. Hence, the characterization of the material refers to macroscopic parameters, such as the flow resistivity and the porosity and to homogenized quantities such as the tortuosity.

A promising approach to manufacture sound absorbers is the application of 3D-printing technologies. 3D-printing allows for the manufacturing of complex shapes directly from Computer-Aided Design (CAD) data. Thereby, the geometries can be designed very freely, since the limitations of conventional manufacturing technologies, e.g., grinding or casting, do not apply. For porous materials, this results in specimen whose microstructure is known and hence can be related to the macroscopic homogenized quantities. In the literature, some examples can be found where 3D-printing technology has been applied to produce sound absorbers. For example, in [17], absorbers are manufactured that apply the principle of destructive interference. Thereby, two connected ducts of different length are produced. The sound waves enter and travel through both ducts separately. The length difference between the ducts produces a phase shift of both traveling waves of 180${}^{\circ }$ at the connection point of the ducts. This leads to a destructive interference effect. However, as the phase shift occurs only for one specific wavelength, the absorption characteristic is rather narrow-banded. This effect is reduced be using several absorbers with different target frequencies together within one sample. Another approach for sound attenuation is shown in [18]. The authors present a structure that reduces sound transmission in ducts while being permeable and thus can be applied within fluid flows. The working principle also employs destructive interference, hence the ability for a high sound transmission loss is available only in a narrow frequency band. An approach that aims for sound absorption in a broader frequency range is presented in [19]. The authors present a multilayered perforated panel absorber and manufacture parts of the structure using 3D-printing technology.

The acoustic properties of porous media can be estimated using several material models of different complexity, among which the Biot-model is the most complex one and seen as the reference [20]. The models estimate the acoustic behavior based on homogenized quantities, such as airflow resistivity, tortuosity, porosity and others. In the following, these are referred to as the Biot-parameters. Using these models, the tailoring of porous media to specific applications becomes possible. For this tailoring process, two prerequisites are necessary. First, the microgeometry of the porous absorber has to be known and can be manufactured with high accuracy. Second, the relation of the microstructure and the Biot-parameters has to be known. This second prerequisite is crucial as it allows the prediction of the acoustic behavior based on the specimen geometry. Nevertheless, this relation is rarely investigated. Within this contribution, the manufacturing and optimization of porous absorbers based on the Biot-model is shown. Thereby, measures are identified that allow for a tailoring of the porous material by adjusting geometric quantities of the investigated specimen. To achieve the first prerequisite, the porous specimen are manufactured using 3D-printing technology. To relate the Biot-parameters to the microstructure, the parameters are inversely estimated from measurements and related to the manufactured geometry. These results are backed up with a sensitivity analysis and an uncertainty quantification in order to obtain the most promising measures for tailoring 3D-printed porous absorbers to specific applications.

## 2. Specimen Description

**Figure 1:**
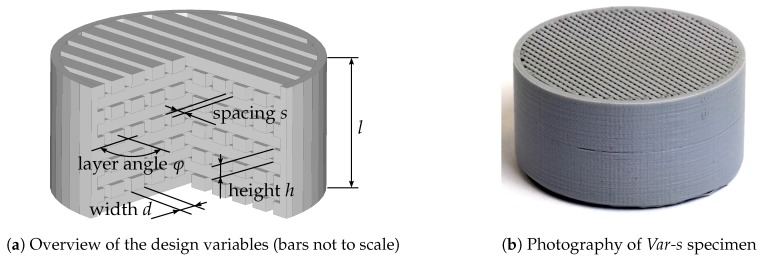
Geometric design (left, CAD model; right, photography) of investigated 3D-printed specimen.

The specimens investigated within this contribution are shown in Figure 1. In Figure 1a, a schematic overview of the construction of the specimen and the investigated design variables is given. The specimens consist of layers with a layer height *h*. Each layer consists of parallel bars with a width *d* and the bars are set apart with a spacing *s*. From one layer to the next, the layers are rotated with an angle φ in clockwise direction. The overall specimen height is denoted with *l*. The specimens are surrounded with a solid ring with a thickness of 1 mm for an increase of the mechanical stability of the specimen and reduction of the leakage in the experimental setups. The top and bottom side of the specimens are left open to allow for the measurement of flow resistivity (see Section 3.1) and absorption coefficient (see Section 3.2) in two directions. In Figure 1b, an image of the specimen with varied bar spacing (*Var-s*) is shown. What can be seen is the last layer of parallel bars and the solid, outer ring. Further, the layered structure that results from the 3D-printing process, can be recognized on the outer surface.

To ensure that the bar structures are separated and no unexpected connections due to manufacturing imperfections occur, following the acoustical tests, the *Ref 2* specimen was cut to provide insight into the specimen cross-section. Figure A3 in the Appendix C presents cross-sectional views of the specimen inner structure. It can be seen that the parallel bars within one layer, as well as the different layers, are separated and no gluing-together of the structures is observed. From these investigations, it is expected that the bar lattice geometry is manufactured according to the specimen design and, hence, the conclusions regarding the influence of the geometry on the Biot-parameters are reasonable.

### 2.1. Geometric Design

Within this contribution, five different configurations were tested: one reference configuration (referred to as *Ref 1/2*) and one variation of each design variable. The geometric dimensions of all variations are given in Table 1. The overall height *l* is 15 mm and the diameter is 30 mm for all specimens. The height was chosen: (a) to reduce the manufacturing time and cost; and (b) to achieve a sufficient thickness so a relevant absorption can be expected in the desired frequency range of 900–6600 Hz. The specimen diameter was chosen according to existing impedance tubes with a diameter of 30 mm that prescribe the investigated frequency range. As shown in the table, two samples of the *Ref 1* configuration were manufactured (*Ref 1/2*) with the same geometric dimensions in order to account for manufacturing uncertainties.

Further, two geometrically equal specimens (*Ref 3/4*) were printed with an overall specimen height *l* of 30 mm. All other geometric dimension are equal to those of specimens *Ref 1/2*. The specimens *Ref 3/4* allow for a verification of the parameter identification process described in Section 4.2.

### 2.2. 3D-Printing Process

The printing of the specimen was done using a standard commercial Ultimaker 3 3D-printer with a fine nozzle. The nozzle diameter is 0.25 mm. The 3D-printer built the entire structure layer by layer, with a layer resolution of 0.15 mm and an xyz-resolution of 12.5, 12.5, 2.5 μm (all technical data according to Ultimaker B.V. [21]). The material used for printing the specimen is Ingeo™Biopolymer 4043D, which is a Polylactic Acid (PLA) Biopolymer [22]. No special treatment was conducted prior to the printing process. The specimen were built up from layers supported on a building platform; the build direction is the vertical direction in Figure 1. Further, no supporting structures were used and, hence, no cleaning or removing of such structures was conducted after the printing process.

## 3. Experimental Characterization

The manufactured specimen were characterized experimentally by measuring the airflow resistivity and the absorption coefficient. As it is expected that the specimen exhibit a direction dependent behavior due to manufacturing imperfections, all measurements were conducted in two directions (vertical direction in Figure 1, in each measurement one of the upper and lower face of the specimen is exposed to the incident waves). The results of the direction dependent measurements were then averaged.

### 3.1. Flow Resistivity Measurements

**Figure 2:**
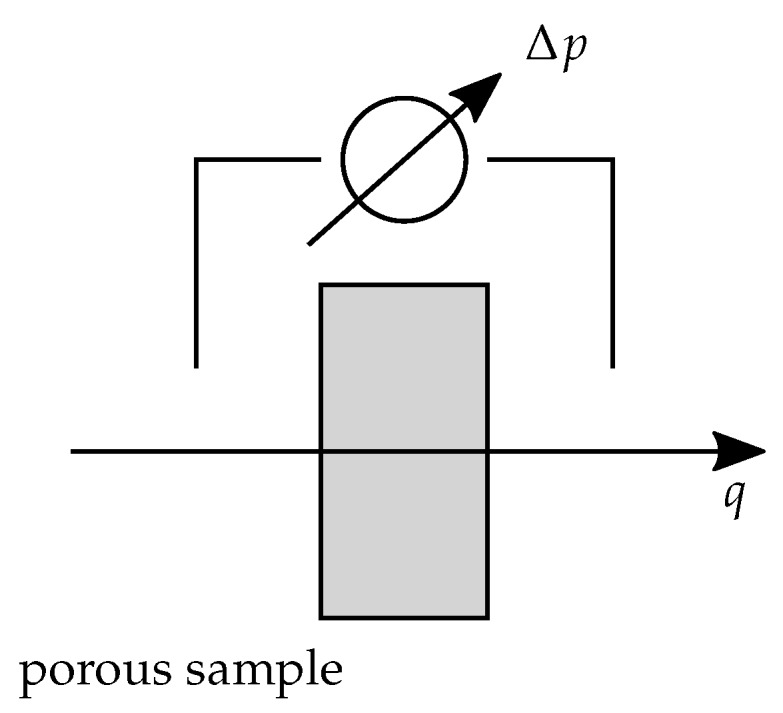
Measurement principle of the flow resistivity measurement.

The measurement of the airflow resistivity was done using the method with alternating airflow, as described in [23,24]. It allows for the determination of the static airflow resistivity of porous media. The measurement principle is to determine the pressure drop Δp over a sample that is subject to a continuous volume flow q=vA. The velocity *v* of the airflow is 0.50 mm/s and the specimen cross-sectional area is denoted by *A*. Thereby, it should be noted that the solid outer ring of the manufactured specimen is neglected during the measurement, the net area that is open to the flow is slightly smaller than the overall specimen cross-sectional area *A*. The measurement principle is shown in Figure 2. Having theses quantities determined, the flow resistance *R* can be determined by R=Δp/q. From the flow resistance *R*, the specific flow resistance $R_{S}$ can be computed as $R_{S}=R\;A$. Using the specimen height *l*, the flow resistivity σ follows as $\sigma =R_{S}/l$. The quantity σ is a material-specific quantity and one of the most important measures for characterizing porous media.

It should be noted that the determination of the flow resistivity involves quite high measurement uncertainties, at least for soft materials such as foams and fibrous materials. In [25,26,27], it is shown that a reproducibility measurement uncertainty of up to ≈15.10% can be expected, whereas the repeatability uncertainty is around 5%. In [28], it is shown that, taking into account the measurement apparatus only, a rather high precision with an uncertainty of around 2% can be expected. Hence, the major part of the uncertainty is caused from imperfections during the specimen manufacturing and installation process. Further, the applied measurement principle with alternating airflow does not account for a direction dependency of the flow resistivity of the specimen as it implicitly gives an averaged quantity. Nevertheless, the specimen was mounted into the measurement apparatus in both directions in order to assure the reproducibility of the measurement.

### 3.2. Absorption Measurements

**Figure 3:**
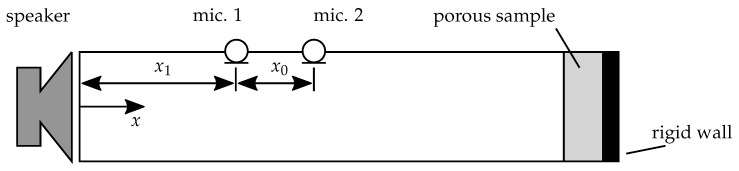
Measurement principle of the absorption coefficient measurement.

The measurement of the absorption coefficient was conducted in an impedance tube with a two-microphone measurement principle according to the method described in [29,30]. A sketch of an impedance tube can be found in Figure 3. Thereby, the porous sample was backed with a rigid wall and, hence, in the tube a standing wave field was excited by the speaker. The sound field was measured using two microphones placed upstream of the sample. Using the transfer function $H_{12}$:

(1) $$H_{12}=\frac{S_{\mathrm{mic}.\;2}\left(\omega \right)}{S_{\mathrm{mic}.\;1}\left(\omega \right)}$$

between the Fourier-transformed signals at the two microphone positions $S_{\mathrm{mic}.\;1}\left(\omega \right)$ and $S_{\mathrm{mic}.\;2}\left(\omega \right)$, respectively, the reflection factor *r* can be determined using the wave number *k*, the microphone distance $x_{0}$ and the distance between the sample and the nearest microphone $x_{1}$. The imaginary unit is denoted by $j=\sqrt{-1}$. The reflection factor is a frequency dependent, complex quantity and can be computed as follows:

(2) $$r=\frac{e^{jkx_{0}}-H_{12}}{H_{12}-e^{-jkx_{0}}}e^{2jkx_{1}}$$

The absorption coefficient α further is derived from the reflection factor with $\alpha =1-{\left|r\right|}^{2}$. It is real valued and frequency dependent. The frequency range that can be used with a given impedance tube is limited for low frequencies by the distance of the microphones $x_{0}$ and for high frequencies by the cut-on frequency. The cut-on frequency is that frequency above which non-planar modes are able to propagate through the tube. The impedance tube can only be used with planar waves, i.e., below the cut-on frequency. The cut-on frequency relates to the speed of sound and the tube diameter. For the presented investigations, a tube with a diameter of 30 mm was used. The distance of the microphones limits the detectable phase difference between the signals, hence, for low frequencies and accordingly high wave lengths, a high microphone distance is necessary. The microphone distance used for the presented measurements was 20 mm. Given the limits for the low and high frequencies, the measurements were conducted for a frequency range of 900–6600 Hz. This range cannot easily be extended as, for measurements in higher frequency regimes, the tube has to be of a smaller diameter and hence would require the manufacturing of new samples of all specimen. This procedure is not feasible, as a new specimen would probably exhibit different material parameters due to uncertainties associated with the production process and thus cannot easily be compared to the existing specimen. Further, it is assumed that the homogeneity of very small specimen lacks due to manufacturing uncertainties. For the low frequency regime an extension of the measurement is possible, but as the measurement results suggest, no relevant absorption is expected due to the rather small specimen thickness of 15 mm.

It should be noted that the presented values for the absorption coefficient are, due to the measurement principle, only valid under plane wave loading, i.e., normal incidence angle. For random incidence angles, different absorption coefficients can be expected. To overcome this difference, for example, in [31,32,33], procedures for computing the random incidence sound absorption coefficient from normal incidence measurements are presented.

## 4. Numerical Characterization

Besides the experimental characterization, the specimens were modeled mathematically using the Biot-model [34,35] and solved numerically using the finite element method (FEM). The goal of this procedure is to inversely extract the Biot-parameters and to relate these to the geometric variations of the specimen described in Section 2.1. The Biot-model assumes the solid phase to be elastic and thus incorporates three wave types traveling within the porous medium, i.e., one shear wave within the solid phase and two compressional waves in both the fluid phase and the solid phase [1]. The consideration of the elasticity of the solid phase distinguishes the Biot-model from the class of models with a rigid solid phase, the so-called complex fluids. This class of models is applicable when the motion of the solid phase can be neglected, i.e., when its stiffness is very high or when the material is mechanically coupled to other components of high stiffness. Well-known material models of this class are, among others, the Champoux–Allard model, the Lafarge et al. model and the Johnson et al. model [36,37]. For the investigations presented in this contribution, the Biot-model seems the most promising one as the material used in the 3D-printer is a thermoplastic with a rather low stiffness. Thus, it is assumed that motions of the solid phase cannot be neglected.

### 4.1. Finite Element Model

**Figure A1:**
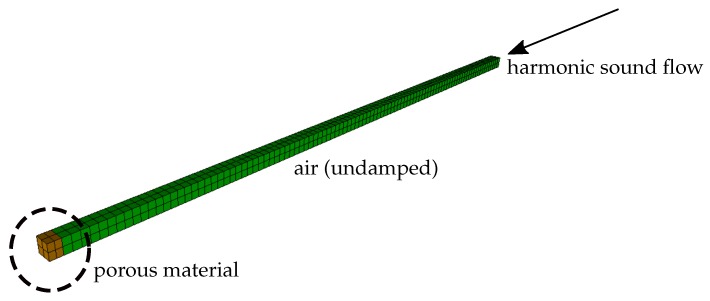
FEM-model of the impedance tube.

**Figure A3:**
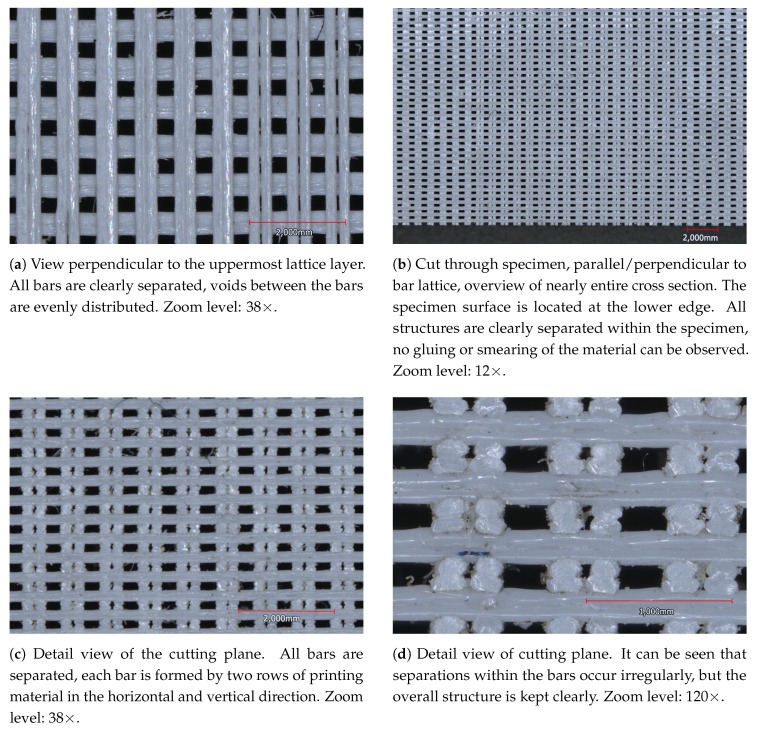
Microscopic cross-sectional views of cut *Ref 2*-specimen.

The numerical characterization was done using a numerical model of an impedance tube, as shown in Figure A1 in the Appendix A. The model represents a generic impedance tube, as shown in Figure 3. The dimensions of the modeled impedance tube were 1 m in length with a square cross section and an edge length of 30 mm. Hence, the cut-on frequency was nearly (difference approximately 15%) identical with the circular impedance tube used for the experiments. The square cross section was used to ease the discretization using hexaeder volume elements. The air within the tube was modeled as an undamped fluid, while the porous material was modeled using the Biot-model. The speaker shown in Figure 3 was modeled using a harmonic sound flow. For both, the air and the porous material, 27-node elements with quadratic ansatz functions were used. The FEM-implementation of the Biot-model uses the four-degree-of-freedom (DOF) u-p-formulation presented in [38,39], i.e., the pore pressure (p) is formulated using the displacements (u) of the solid phase. It should be noted here that the motivation for applying the Biot-model instead of modeling the microstructure of the material directly is to allow for an application of the obtained parameters in a realistic scenario, where the modeling of the microstructure is prohibitively expensive and homogenized material models are necessary.

The element edge length of all elements was 10 mm and thus allowed the application of the discretization up to a frequency of 7555 Hz with a resolution of ten nodes per wavelength. Hence, a high accuracy of the computed sound pressure distribution within the tube can be expected. The resulting finite element model consisted of 412 elements and resulted in a system of equations with a total of 5500 DOF. The system of equations was solved in the frequency domain using the in-house code ePaSo (in-house code of the Institute for Acoustics, TU Braunschweig; see https://www.tu-braunschweig.de/ina/tech/elpaso/index.html) [40]. The rather small dimension of the computational problem allowed for a fast computation of the sound pressure distribution and thus enabled the inverse parameter identification using an optimization strategy, as described in Section 4.2.

The absorption coefficient is computed from the sound pressure distribution along the central axis of the impedance tube using the so called mini-max procedure, described for example in [41]. The principle computes the absorption coefficient from the sound pressure distribution p(x), where the coordinate *x* denotes the direction along the impedance tube axis.

(3) $$\begin{array}{cc} \alpha & =\frac{2}{1+\frac{1}{2}\left(\mu +\frac{1}{\mu }\right)}\\ \mathrm{with}\\ \mu & =\frac{\min\left(p(x)\right)}{\max\left(p(x)\right)} \end{array}$$

### 4.2. Inverse Parameter Identification

The inverse parameter identification is based upon the minimization of the mean squared difference between the measured absorption coefficient $\alpha^{M}$ and the computed value $\alpha^{C}$. Thereby, the solution $\alpha^{C}$ was computed in the frequency domain for 218 frequency steps in the range of 900–6600 Hz with a frequency step size of 30 Hz. The much higher resolved measured absorption coefficient $\alpha^{M}$ was interpolated to the frequency steps used in the computation. The target function Γ to be minimized is given as follows:

(4) $$\Gamma \left(\alpha_{\infty },\phi ,\Lambda ,\Lambda^{\prime }\right)=\frac{1}{n}\sum_{i=1}^{n}{\left(\alpha_{i}^{M}-\alpha_{i}^{C}\right)}^{2}$$

The computed absorption coefficient and thus the target function depends on the material parameters of the Biot-model, as given in Table 2. The mechanical properties for the solid phase were taken from the 3D-printing material data sheet [22], while the values for the Poisson’s ratio and the damping were based on empirical values. The air density was computed based on the room temperature using the ideal gas law. The flow resistivity was measured for all specimens using the procedure described in Section 3.1. Hence, the remaining parameters for the optimization procedure are the *tortuosity* $\alpha_{\infty }$, the *porosity* ϕ, the *viscous characteristic length* Λ and the *thermal characteristic length* $\Lambda^{\prime }$. The minimization procedure was implemented using the Nelder–Mead simplex algorithm [42] taken from the Python library *scipy* [43]. The optimization was, due to the used algorithm, set up as an unconstrained optimization problem. Nevertheless, as negative values are not meaningful for the modeled physical quantities, only positively valued results were accepted as a sufficient result. It should be noted that using the initial parameter set given in Table 2, all inversely identified parameters were positive for every run of the optimization.

The presented inverse parameter identification procedure is similar to the one described in [44]. Nevertheless, in this contribution, the applied material model is the full Biot-model, whereas in [44] a formulation based on the Johnson et al. and the Champoux-Allard model is applied. Further, within the procedure presented here, the porosity was evaluated using the optimization technique as well, whereas in [44], the porosity is measured directly.

**Figure 4:**
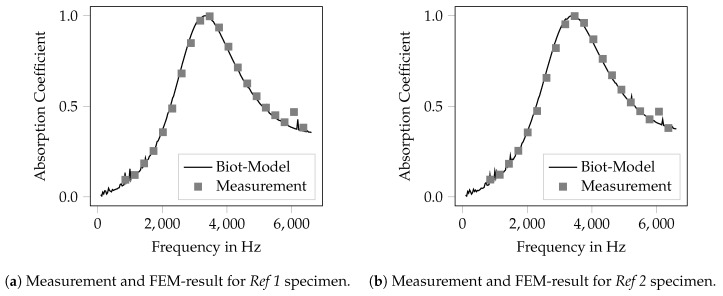
Measured and computed absorption characteristics after inverse parameter identification *Ref 1/2* specimen.

The inverse parameter identification was run for each specimen given in Table 1, the identified Biot-parameters are given in Table 3. In Figure 4, the results are shown for the specimen *Ref 1* (Figure 4a) and *Ref 2* (Figure 4b). It can be seen that the computed absorption coefficient fits the measured data fairly well. This holds as well for all other investigated specimens. The results of the other specimens are shown graphically in Appendix B. Previous studies of the authors on well-known melamine foams and the works in [44] have shown the ability of the presented method to obtain physically reasonable results and based on the good agreement of the measured and the computed absorption coefficient, it can be expected that the identified material parameters represent a good estimation of the physically correct values. Nonetheless, the optimization problem might not be unique, thus the presented optimal result might not be the global optimum.

In Table 3, the inversely identified parameters for the Biot-model are shown for all investigated specimens. Further, for each quantity, the relative deviation from the *Ref 1* configuration is given. It can be observed that for all specimens the viscous and thermal characteristic lengths are nearly equal, whereas the thermal characteristic length is slightly smaller than the viscous characteristic length. This is contradictory to the findings in [44] after that the viscous length typically is smaller than the thermal length. Nevertheless, the difference between both is small, therefore it is assumed that no general error occurred during the parameter identification. Further, the estimated porosity is near [round-precision=1].4. As the manufactured specimen are regular and periodic structures, the porosity can be estimated analytically using the bar spacing and width. Assuming an infinitely extended lattice in the layer plane and using the dimension given in Table 1, the theoretical porosity is .375. Thus, the inversely identified parameters seem reasonable and were used in the following investigations.

The specimen *Ref 1* and *Ref 2* were printed using the same CAD-file and hence should show equal Biot-parameters. Nevertheless, a difference of up to 7.4% can be observed for flow resistivity. The other, numerically identified parameters differ less, with up to 4.0%. The variation of the design variables also led to altered Biot-parameters. It can be seen that the variation of the layer angle thereby results in the largest variations of the inversely identified parameters.

### 4.3. Verification of the Inversely Identified Parameters

To allow for a verification of the inversely identified parameter sets, the following procedure was chosen: it is expected that the set of Biot-parameters gives a description of the porous material. Hence, a variation of the macroscopic geometric dimensions of the specimen (length and diameter) does not affect the material parameters. Accordingly, specimens with other lengths than the ones used for the parameter identification can be described using the same Biot-parameters. For this investigation, two specimens (*Ref 3/4*) with an overall length of l=30mm were printed and their absorption coefficient was measured. The microscopic dimensions of the bar lattice of both specimen are equal to specimens *Ref 1/2*.

**Figure 5:**
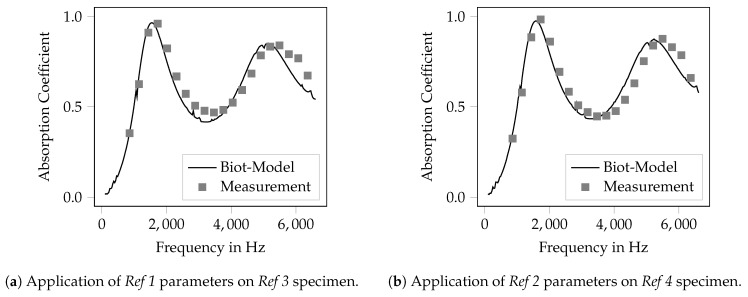
Verification of the inverse parameter identification by applying the identified Biot-parameters to specimen with l=30mm.

In Figure 5, the measured absorption coefficient for the verification samples with a specimen length of 30 mm is shown. Moreover, the computed absorption coefficient is given. The computational result was produced using the FEM-model of the same impedance tube as was used for the parameter identification, but the porous sample was geometrically modeled with the varied overall length of 30 mm. The material model used for the shown computations was parameterized using the parameter values from the *Ref 1/2* configuration given in Table 3. It can be seen that the difference of the computed and measured results is rather low. Within the frequency domain, two absorption maxima are present. Both are met fairly well regarding the frequency and the maximum absorption. As expected, the frequency of the absorption maximum drops from 3370 Hz (15 mm) to 1600 Hz for the 30 mm long specimen. For the second absorption maximum, the frequency of the peak was computed at a slightly lower value than it appears in the measurement. Nevertheless, the value of the maximum absorption is met quite well, whereas the parameters of the *Ref 2* configuration fit the measured data slightly better.

In total, this investigation shows that the inversely identified Biot-parameters are valid for a given set of microscopic dimensions of the bar lattice and remain valid if the macroscopic specimen dimensions are altered. Hence, the rather narrow absorption maximum that can be seen in Figure 4 cannot be attributed to a resonance effect but is the effect of a porous material.

### 4.4. Uncertainty Quantification

The investigations on specimens *Ref 1/2* show a rather good reproducibility of the manufacturing process. Nevertheless, there are uncertainties associated with the produced specimen geometry and, hence, the Biot-parameters become uncertain as well. To account for these uncertainties, an uncertainty quantification was conducted to get an impression of the influence of parameter variations due to manufacturing uncertainties. The uncertainty quantification was conducted as a one-at-a-time parameter variation based on the analytic procedure of the *Guide to the expression of uncertainty in measurement (GUM)*, described in [45,46]. The procedure computes the combined uncertainty $u_{c}$ of the quantity of interest (here, the absorption coefficient) as the sum of the squared product of the sensitivity coefficient $c_{i}$ and the uncertainty of the input parameter $x_{i}$, as shown in Equation (5). The sensitivity $c_{i}$ is the partial derivative of the absorption coefficient after all input parameters, i.e., the Biot-parameters.

(5) $$\begin{array}{cc} u_{c}^{2} & =\sum_{i=1}^{N}{\left[c_{i}u\left(x_{i}\right)\right]}^{2}\\ \mathrm{with}\\ c_{i} & ={\left.\frac{\partial \alpha }{\partial x_{i}}\right|}_{x_{i}}\\ \mathrm{and}\\ x_{i} & \in \left[\alpha_{\infty },r,\phi ,\Lambda ,\Lambda^{\prime }\right] \end{array}$$

For the uncertainty quantification, an uncertainty of 10% of the mean value given in Table 3 for all input parameters was assumed. The uncertainty quantification was done exemplarily for the *Ref 3* configuration. The derivative in the sensitivity coefficient given in Equation (5) was computed numerically using the central differential quotient:

(6) $$c_{i}={\left.\frac{\partial \alpha }{\partial x_{i}}\right|}_{x_{i}}\approx \frac{{\left.\alpha \right|}_{x_{i}+\Delta x_{i}}-{\left.\alpha \right|}_{x_{i}-\Delta x_{i}}}{2\Delta x_{i}}.$$

Thus, for each parameter, a parameter variation was done, the sensitivity was computed and all uncertainty contributions were combined to the combined uncertainty. The sensitivity coefficients are further used in the investigation presented in Section 5 for determining possible design variables for an optimization of the material. It should be noted that this procedure is only valid for small perturbations around the mean value and the resulting sensitivities are only valid at the evaluation point because the underlying function can not be assumed to be linear. These drawbacks can be overcome using a global sensitivity analysis, for example using the Saltelli-method as described in [47]. In [48], a global sensitivity analysis is presented for the Johnson–Champoux–Allard-model using a transfer-matrix-method implementation modeling a three-layer NFRP (natural fiber reinforced plastic) component. Further, global sensitivity analysis on different material models for porous media is carried out in [49]. Nevertheless, the global sensitivity analysis is based on a Monte-Carlo method and thus is computationally very costly. This especially holds for the FEM-model used in this contribution, therefore the one-at-a-time parameter variation was used as described before.

**Figure 6:**
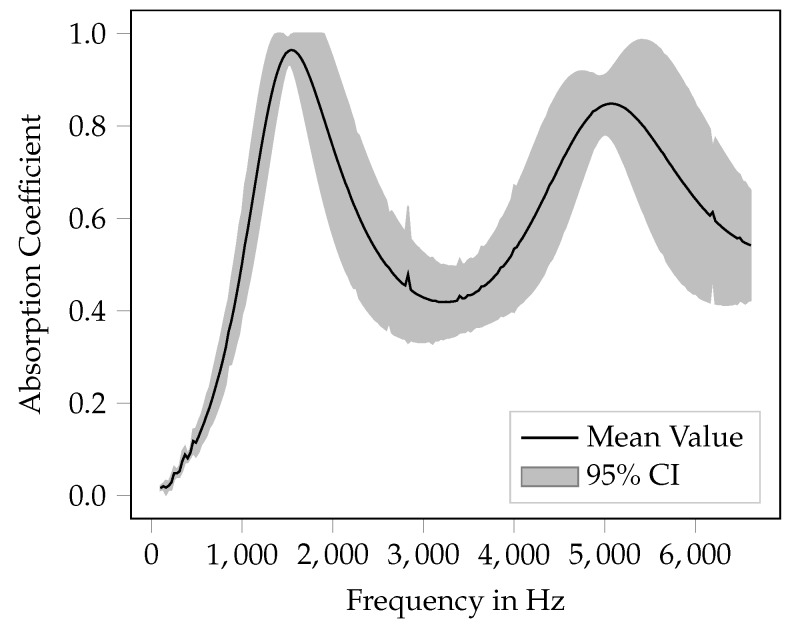
Mean value and confidence interval (CI) for *Ref 3* specimen (10% variation of all input parameters).

The result of the uncertainty quantification is shown in Figure 6. In the figure, the mean absorption coefficient of the *Ref 3*-specimen is plotted as a solid line. Further, the 95% confidence interval is shown. The confidence interval (1.96 times the standard deviation) is computed from the combined uncertainty $u_{c}$, which represents one standard deviation. It can be seen that, for uncertain input parameters, the absorption coefficient is mainly affected in the frequency domain above the first absorption maximum, whereas the effect is rather low in the lower frequency domain. In general, the effect of uncertain parameters grows with the frequency, except from the point where the highest absorption occurs. Here, the effect of uncertainty is very low.

## 5. Optimization of the Porous Material

**Figure 7:**
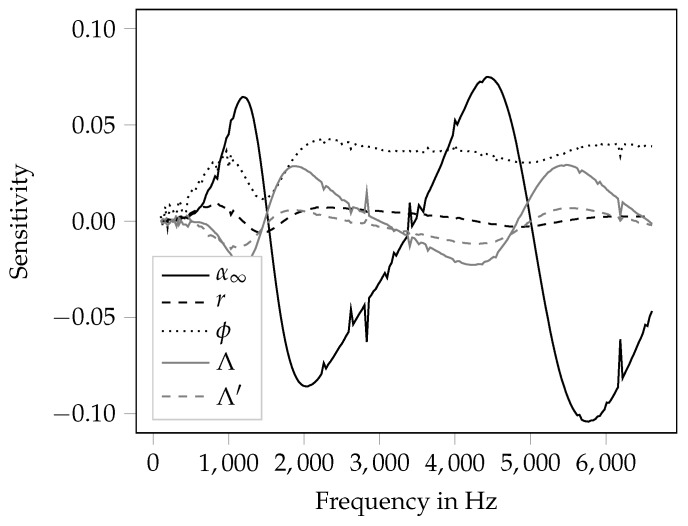
Frequency-dependent sensitivity coefficients of all input parameters.

The overall aim of the presented work is to investigate the possibility of optimizing the printed porous material for a good sound absorption characteristic. It is assumed that a good absorption characteristic incorporates high absorption coefficients in a broad frequency band. In Table 3, it can be seen that the variation of the rotation angle of the layers results in a high variation of the Biot-parameters. Thereby, the flow resistivity, the tortuosity and the thermal characteristic length are the parameters that are affected most by rotating the bar layers. On the other side, a sensitivity analysis on the Biot-parameters can give insight in how the direct variation of theses values influences the absorption coefficient. In Figure 7, the term $\left[c_{i}u\left(x_{i}\right)\right]$ from Equation (5) is plotted for all inversely identified parameters over the frequency. The reason for plotting the product of sensitivity coefficient $c_{i}$ and the related uncertainty $u\left(x_{i}\right)$ is that the term becomes dimensionless and thus the different values can be compared directly. For all input parameters with values of the sensitivity term greater than zero, an increase of the parameter leads to a higher absorption coefficient, whereas a negative sensitivity value indicates a decrease of the absorption coefficient with an increase of the parameter.

What can be observed in Figure 7 is that most quantities have a frequency dependent influence on the absorption coefficient. Generally, the tortuosity has the highest influence on the absorption coefficient. This is somewhat contradictory to the findings in [49], where the flow resistivity is identified as the most influential parameter. Thereby, the negative values in the frequency ranges 1540–3490 Hz and 5020–6610 Hz indicate that an increase of the tortuosity leads to a reduction of the absorption coefficient. In the other frequency ranges, increasing the tortuosity increases the absorption coefficient as well. This behavior is generally apparent for all Biot-parameters except from the porosity, which shows positive sensitivities in the entire frequency range. Hence, the increase of the porosity is beneficial in the entire frequency range. In general, for an optimization of the material with the goal of a broadband increase of the absorption coefficient, design variables that exhibit the same direction of the sensitivity in the entire frequency range are most handy. For the investigated material in this contribution, only the porosity fulfills this requirement. The flow resistivity generally has a rather low sensitivity, but the sensitivity is positive in nearly the entire frequency range. Only at the absorption maxima at 1600 Hz and 5200 Hz weak negative sensitivities can be observed. Summarizing, for increasing the absorption coefficient in a broadband sense, an increase of the porosity and of the flow resistivity is beneficial.

Based on the observation that both the porosity and (except from very narrow frequency bands) the flow resistivity show a sensitivity that has no change of sign in the entire frequency range, these parameters can be used for an optimization of the porous material. Nevertheless, as shown in Table 3, the porosity is not affected strongly by a variation of the geometric parameters of the material. On the other side, the flow resistivity can easily be adjusted using the layer angle of the material. Hence, this parameter can be optimized in order to obtain a high absorption coefficient in a broad frequency range. Therefore, an optimization strategy was set up to maximize the absorption coefficient by adjusting the flow resistivity of the specimen. Nevertheless, the variation of the rotation angle of the layers results in changes of the other Biot-parameters as well, especially the tortuosity. Thus, this approach is a somewhat pragmatic procedure and might not be able to produce optimal results. As the target function to be minimized, the inverse of the sum of the squared frequency dependent absorption coefficients was used:

(7) $$\Gamma \left(\sigma \right)=\frac{1}{\sum_{i=1}^{N}\alpha_{i}^{2}}$$

**Figure 8:**
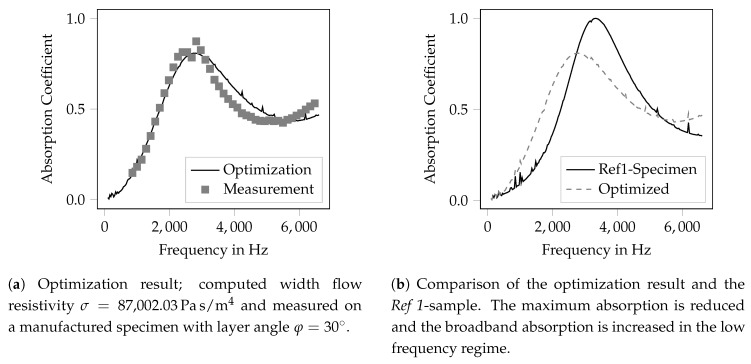
Result of the material optimization by variation of the layer angle.

For the optimization procedure, the Biot-parameters of the *Var-φ* specimen were used and the flow resistivity was optimized. Based on the target function (Equation (7)), the optimal value of the flow resistivity is $\sigma_{opt}\approx 120.000\mathrm{Pa}s/m^{4}$. As there is no direct link between the flow resistivity and the layer angle, this information cannot be translated directly into the necessary layer angle. Instead, two further specimen were manufactured with layer angles of 60${}^{\circ }$ and 30${}^{\circ }$, respectively. All other geometric parameters were kept identical to those of the angle variation specimen. For both samples, the flow resistivity was measured. The material with a layer angle of 60${}^{\circ }$ shows a flow resistivity of $\sigma_{60{}^{\circ }}=78,603.28\mathrm{Pa}s/m^{4}$, the specimen with an angle of 30${}^{\circ }$ has a flow resistivity of $\sigma_{30{}^{\circ }}=87,002.03\mathrm{Pa}s/m^{4}$. However, neither specimen shows the desired flow resistivity that is obtained using the optimization procedure, but they get quite close. The optimization result is summarized in Figure 8 by a comparison to the measured absorption coefficient and to the *Ref 1* sample, respectively.

In Figure 8a, the measured absorption coefficient of the specimen with the layer angle of 30${}^{\circ }$ and the resulting absorption coefficient from the optimization procedure is shown. It can be seen that in the frequency range below the peak the computed result meets the measurement quite well. Nevertheless, in the frequency range above the highest absorption coefficient, some differences can be observed. These can be attributed to changes of the other Biot-parameters that are affected from the variation of the layer angle as well. Further, as the flow resistivity is changed quite dramatically compared to the small perturbations used for the sensitivity analysis, it must be expected that the linearity-requirement is not fulfilled.

The comparison to the specimen *Ref 1* is shown in Figure 8b. It can be seen that the maximum of the absorption coefficient drops from 0.99 (*Ref 1*) to 0.81 (optimized geometry). Meanwhile, the overall magnitude of the absorption coefficient is increased, especially below 3000 Hz and above 5800 Hz. It should be noted that the mean absorption coefficient of both specimen are nearly equal with $\alpha_{\mathrm{Ref}\;1\text{-}\mathrm{mean}}=0.487$ and $\alpha_{\mathrm{Opt}\text{-}\mathrm{mean}}=0.492$. Hence, the optimization does not result in a significant increase of the absorption coefficient, but the areas of high absorption are more evenly distributed along the frequency axis. This may be the result of the chosen target function (Equation (7)). Nevertheless, a more homogeneous distribution of the absorption characteristics can be of more practical use than the narrow banded absorption characteristics of the *Ref 1* sample; hence, the optimization outcome is assumed to be suitable for practical needs. It can be summarized that the adjustment of the flow resistivity indeed leads to an improved acoustical behavior but that it is not the only quantity of interest for a suitable tailoring of porous media.

## 6. Conclusions and Outlook

The application of porous material is a common and widely applied noise mitigation measure. The most common application is the adjustment of room acoustics using porous absorbers; another application is the reduction of aeroacoustic sources. Porous materials typically exhibit a broadband absorption characteristic with an absorption coefficient that generally increases with frequency. The characterization of porous materials oftentimes relies on the measurement of macroscopic quantities, such as the flow resistivity. Further, homogenized values, i.e., the Biot-parameters porosity, tortuosity and characteristic lengths, are needed to use material models such as the Biot-model and models from the class of complex fluids. As the geometric structure on the microscale of the porous materials is generally unknown, the Biot-parameters cannot be related directly to the geometric structure. Hence, tailoring the material to a desired acoustic behavior by adjusting the material geometry on the microscale is not possible yet.

In this contribution, generic porous materials were manufactured based on lattices of bars. The dimensions of these bars served as design variables for the design of the porous material. Each bar lattice formed one layer of the porous material, several layers were stacked above each other to build the entire specimen. Thereby, the layers were rotated around a specified angle from one layer to the next. The material was manufactured using 3D-printing technology. Thereby, parameter variations of all geometric design variables were investigated. Using inverse parameter identification based on the Biot-model and a finite-element formulation, the Biot-parameters were identified from measurements of the absorption coefficient. It can be shown that most design variables have a rather small influence on the Biot-parameters, whereas the layer angle strongly influences the flow resistivity. Further, it can be shown numerically that the porosity and the flow resistivity both have a positive sensitivity regarding the absorption coefficient in the entire frequency range. Hence, these parameters can be used for an optimization of the material. Together with the observation that the flow resistivity is strongly affected by the layer angle, the material is optimized based on a given set of Biot-parameters and an optimization of the flow resistivity. It can be shown that this optimal flow resistivity can be accomplished effectively by an adjustment of the layer angle of the specimen.

Summarizing, the presented procedure introduces a possible methodology to generate tailored porous materials based on an optimized set of Biot-parameters. It can be shown that for generic specimen the Biot-parameters can be connected to the geometric design of the specimen and, thus, an optimization of the material on the microscale becomes possible. Moreover, it can be shown that 3D-printing technology is a promising approach for manufacturing porous materials, as the degrees of freedom during the printing process allow for the manufacturing of complex structures that are needed for porous material design.

During the presented procedure, it is necessary to optimize the material with respect to the flow resistivity. To connect the flow resistivity with the design variables, specimen with an estimated set of design variables are manufactured and the flow resistivity is measured. This procedure can be done using numerical methods as well by applying computational fluid dynamics (CFD) methods for estimating the flow resistivity. This procedure is shown for 3D-printed specimen by the authors in [50].

Future work is planned on increasing the porosity of the printed specimen, as this parameter shows an even higher sensitivity as the flow resistivity and thus is a promising approach to further increase the absorption characteristic of the presented specimen. Another part of the work aims for an estimation of the remaining Biot-parameters from the geometry.

## Figures and Tables

**Table 1:** Overview of the investigated variations.

Name	Layer Height *h* in mm	Bar Width *d* in mm	Bar Spacing *s* in mm	Rotation Angle φ in ${}^{\circ }$	Specimen Height *l* in mm
*Ref 1/2*	0.20	0.50	0.30	90.0	15.00
*Ref 3/4*	0.20	0.50	0.30	90.0	30.00
*Var-h*	0.30	0.50	0.30	90.0	15.00
*Var-d*	0.20	0.60	0.30	90.0	15.00
*Var-s*	0.20	0.50	0.40	90.0	15.00
*Var-φ*	0.20	0.50	0.30	45.0	15.00

**Table 2:** Material parameters for Biot-model and data sources before optimization.

Parameter	Variable	Phase	Value	Dimension	Data Source
Young’s Modulus	*E*	solid	3.6 × 10${}^{6}$	Pa	data sheet [22]
Skeleton Density	$\rho_{S}$	solid	1240	$kg/m^{3}$	
Poisson’s ratio	ν	solid	0.40	–	empirical value
Damping coefficient	η	solid	0.10	–	empirical value
Air Density	$\rho_{F}$	fluid	1.225	$kg/m^{3}$	from ideal gas law
Flow resistivity	σ	fluid	see Table 3	Pas/m4	measured
Tortuosity	$\alpha_{\infty }$	solid	2.00	–	initial guess
Porosity	ϕ	solid	0.40	–	
Viscous characteristic length	Λ	solid	1 × 10${}^{-4}$	μm	
Thermal characteristic length	$\Lambda^{\prime }$	solid	1 × 10${}^{-4}$	μm	

**Table 3:** Overview of inversely identified material parameters and variation relative to *Ref 1* specimen.

Specimen/Variation	Tortuosity $\alpha_{\infty }$		Flow Resistivity σ in Pa s/m${}^{4}$		Porosity ϕ		Viscous Char. Length Λ in μm		Thermal Char. Length $\Lambda^{\prime }$ in μm	
*Ref 1*	1.96	0.0%	21,750.03	0.0%	0.41	0.0%	105.04	0.0%	100.82	0.0%
*Ref 2*	1.88	4.0%	20,145.09	7.4%	0.42	3.9%	103.31	1.7%	101.93	1.1%
*Var d*	1.59	19.0%	10,620.08	51.2%	0.43	5.4%	108.92	3.7%	105.15	4.3%
*Var s*	2.04	4.3%	22,140.18	1.8%	0.39	3.0%	99.77	5.0%	100.55	0.3%
*Var h*	1.76	10.0%	19,230.11	11.6%	0.42	3.8%	107.86	2.7%	102.23	1.4%
*Var φ*	2.68	36.7%	53,851.62	147.6	0.40	0.9%	80.71	23.2%	84.77	15.9%

**Figure A2:**
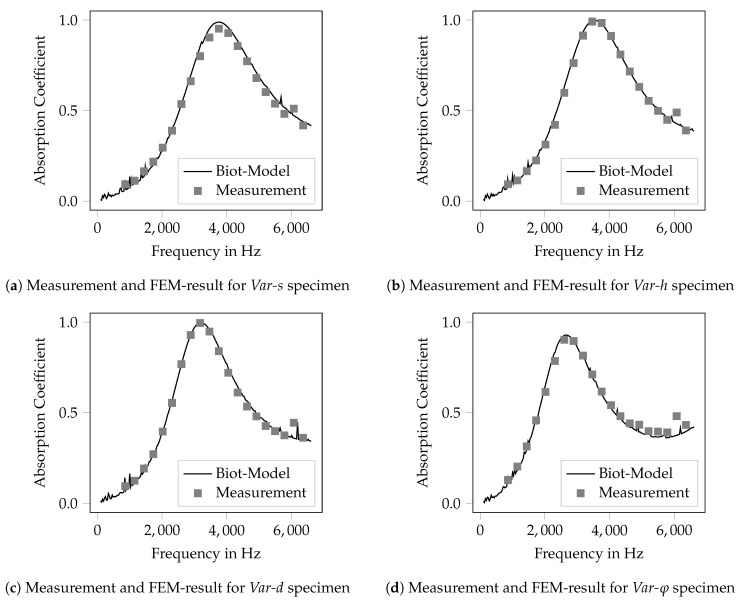
Further results for measured and computed absorption characteristics after inverse parameter identification.

## Abbreviations

The following abbreviations are used in this manuscript:

CAD	Computer-Aided Design
CRC	Collaborative Research Center
CFD	Computational Fluid Dynamics
DOF	Degree of freedom
elPaSo	elementary Parallel Solver
FEM	Finite-Element Method
GUM	Guide to the Expression of Uncertainty in Measurement
PLA	Polyactic Acid
*A*	specimen cross-sectional area
$c_{i}$	sensitivity coefficient of *i*th input parameter
*d*	bar width
*E*	Young’s Modulus
*h*	layer height
$H_{12}$	transfer function between microphone 1 and microphone 2
*j*	imaginary unit $\left(\sqrt{-1}\right)$
*k*	wave number
*l*	specimen height
*q*	volume flow through the specimen
*r*	reflection factor
*R*	flow resistance
$R_{S}$	specific flow resistance
*s*	bar spacing
$u_{c}$	combined uncertainty
*v*	flow velocity through the specimen
*x*	axial coordinate in the impedance tube
$x_{0}$	microphone distance
$x_{1}$	distance from microphone 1 to the specimen
$x_{i}$	i-th input quantity
α	absorption coefficient (superscripts: *M*, measured; *C*, computed)
$\alpha_{\infty }$	tortuosity
$\Gamma \left(\ldots \right)$	target function to be optimized
Δp	pressure drop over the specimen
η	damping coefficient
Λ	viscous characteristic length
$\Lambda^{\prime }$	thermal characteristic length
ν	Poisson’s ratio
$\rho_{F}$	air density
$\rho_{S}$	skeleton density
σ	flow resistivity
φ	layer rotation angle
ϕ	porosity
